# Results of the extended analysis for cancer treatment (EXACT) trial: a prospective translational study evaluating individualized treatment regimens in oncology

**DOI:** 10.18632/oncotarget.26604

**Published:** 2019-01-29

**Authors:** Gerald W. Prager, Matthias Unseld, Fredrik Waneck, Robert Mader, Fritz Wrba, Markus Raderer, Thorsten Fuereder, Phillip Staber, Ulrich Jäger, Markus Kieler, Daniela Bianconi, Mir Alireza Hoda, Lukas Baumann, Alexander Reinthaller, Walter Berger, Christoph Grimm, Heinz Kölbl, Maria Sibilia, Leonhard Müllauer, Christoph Zielinski

**Affiliations:** ^1^ Department of Medicine I, Division of Oncology, Medical University of Vienna, Vienna, Austria; ^2^ Clinical Institute of Pathology, Medical University of Vienna, Vienna, Austria; ^3^ Department of Interventional Radiology, Medical University of Vienna, Vienna, Austria; ^4^ Department of Medicine I, Division of Hematology and Hemostaseology, Medical University of Vienna, Vienna, Austria; ^5^ Department of General Gynecology and Gynecological Oncology, Medical University of Vienna, Vienna, Austria; ^6^ Department of Medicine I, Institute of Cancer Research, Medical University of Vienna, Vienna, Austria; ^7^ Department of Surgery, Institute of Cancer Research, Medical University of Vienna, Vienna, Austria; ^8^ Comprehensive Cancer Center of the Medical University of Vienna, Vienna, Austria; ^9^ Center for Medical Statistics, Informatics, and Intelligent Systems, Medical University of Vienna, Vienna, Austria

**Keywords:** precision medicine, molecular profile

## Abstract

**Background:**

The concept of personalized medicine defines a promising approach in cancer care. High-throughput genomic profiling of tumor specimens allows the identification of actionable mutations that potentially lead to tailored treatment for individuals’ benefit. The aim of this study was to prove efficacy of a personalized treatment option in solid tumor patients after failure of standard treatment concepts.

**Results:**

Final analysis demonstrates that 34 patients (62%) had a longer PFS upon experimental treatment (PFS1) when compared to previous therapy (PFS0); PFS ratio > 1.0 (*p* = 0.002). The median PFS under targeted therapy based on molecular profiling (PFS1) was 112 days (quartiles 66/201) and PFS0 = 61 days (quartiles 51/92; *p* = 0.002). Of the 55 patients, 31 (56%) showed disease control (DCR), consisting of 2 (4%) patients which achieved a complete remission, 14 (25%) patients with a partial remission and 15 (27%) patients who had a stabilization of disease. Median OS from start of experimental therapy was 348 days (quartiles 177/664).

**Conclusion:**

The prospective trial EXACT suggests that treatment based on real-time molecular tumor profiling leads to superior clinical benefit.

**Materials and Methods:**

In this prospective clinical phase II trial, 55 cancer patients, after failure of standard treatment options, aimed to achieve a longer progression-free survival on the experimental treatment based on the individual’s molecular profile (PFS1) when compared to the last treatment given before (PFS0). The personalized medicine approach was conceived to be clinical beneficial for patients who show a PFS ratio (PFS 1/PFS0) of > 1.0.

## INTRODUCTION

Etiological concepts on cancer development, malignant growth and tumor propagation have led to the discovery of various molecular driver mechanisms [1, 2]. Based on these advances, medical oncology has started to enter an era of individualized medicine where treatment selection is becoming tailored to druggable molecular targets [1–3]. This individualized treatment concept is mainly based on molecular and genetic characterization of the tumors including next generation sequencing (NGS), which allows to align the most appropriate treatment according to the patient’s disease [4]. Although there is a general acceptance towards such individualized approach, thereby stratifying and subgrouping patients to improve the quality of clinical care in oncology, molecular profiling has just started to assist prediction of the drug’s clinical benefit by identifying the most responsive patient subgroup. Specific molecules, such as EGFR, KRAS, BRAF, estrogen receptor, HER2, etc. are already associated with patient populations that respond more likely to target therapy [5–8]. However, most genes have been largely constrained to organ-specific analysis (e.g. estrogen receptor /HER2 for breast cancer, KRAS for colorectal cancer, BRAF for melanoma, etc.). Recently, molecular profile-based therapy has shown clinical benefit in different cancer types independent of their primary tumor site of origin [2, 9, 10]. Such a tailored treatment strategy revealed to be an effective approach to increase progression free survival (PFS), when compared to the patients’ most recent standard treatment regimen.

Here we aimed to verify the concept of individualized therapy by using molecular profiling of patient tumors in the era of immunotherapies. We could demonstrate that in certain patients an individualized treatment upon a real-time assessment of the tumor’s molecular profile might reflect an efficient strategy to control the disease.

## RESULTS

### Patient characteristics and study algorithm

Patients with metastatic solid tumors after failure of any standard treatment options were real-time biopsated and fresh tumor material was investigated for possible druggable targets via NGS, IHC and FISH. Results of the molecular profile were discussed by a multidisciplinary team for treatment decision. From 114 patients screened, 55 patients (48%) were eligible to start treatment upon the molecular profile derived from real-time biopsy. 35 (64%) men and 20 (36%) women with a mean age of 61 years (± 14) where included. At time of censoring (12/31/2016), 27 patients (48.2%) were still alive while 29 (51.8%) patients were deceased. Most common tumor types analyzed were colon cancer (*n =* 7; 12.3%), cholangiocellular cancer (*n =* 6; 10.5%), head and neck cancer (*n =* 5; 8.8%), thyroid cancer (*n =* 5; 8.8%) and lymphomas (*n =* 4; 7%) (Table 1).

**Table 1:** Patient characteristics and tumor types in EXACT

Gender	*n*	%
Male	35	63.6
Female	20	36.4
Age	years	Range
	61	23-84
	***n***	**%**
Alive	27	49.1
Dead	28	50.9
**Tumor type**	***n***	**%**
CRC	7	12.3
CCC	6	10.5
Head & neck cancer	5	8.8
Thyroid cancer	5	8.8
Lymphoma	4	7.0
Mesothelioma	3	5.3
CUP	3	5.3
HCC	2	3.5
Esophageal cancer	2	3.5
PEComa (renal)	2	3.5
Ovarian cancer	2	3.5
PDAC	2	3.5
NET	2	3.5
Adrenal cancer	1	1.8
Hepatoid Peritoneum	1	1.8
Vulvar carcinoma	1	1.8
Breast cancer	1	1.8
Prostate cancer	1	1.8
Urothelial cancer	1	1.8
Testicular cancer	1	1.8
Multiple Myeloma	1	1.8
Endometrial cancer	1	1.8
Melanoma	1	1.8
Total	55	100.0%

CRC: colorectal cancer; CCC: cholangiocarcinoma; CUP: cancer of unknown primary; HCC: hepatocellular carcinoma; PEComa: perivascular epithelioid cell tumor of the kidney; PDAC: pancreatic ductal adenocarcinoma; NET: neuroendocrine tumor.

### Molecular profile

**Figure 1:**
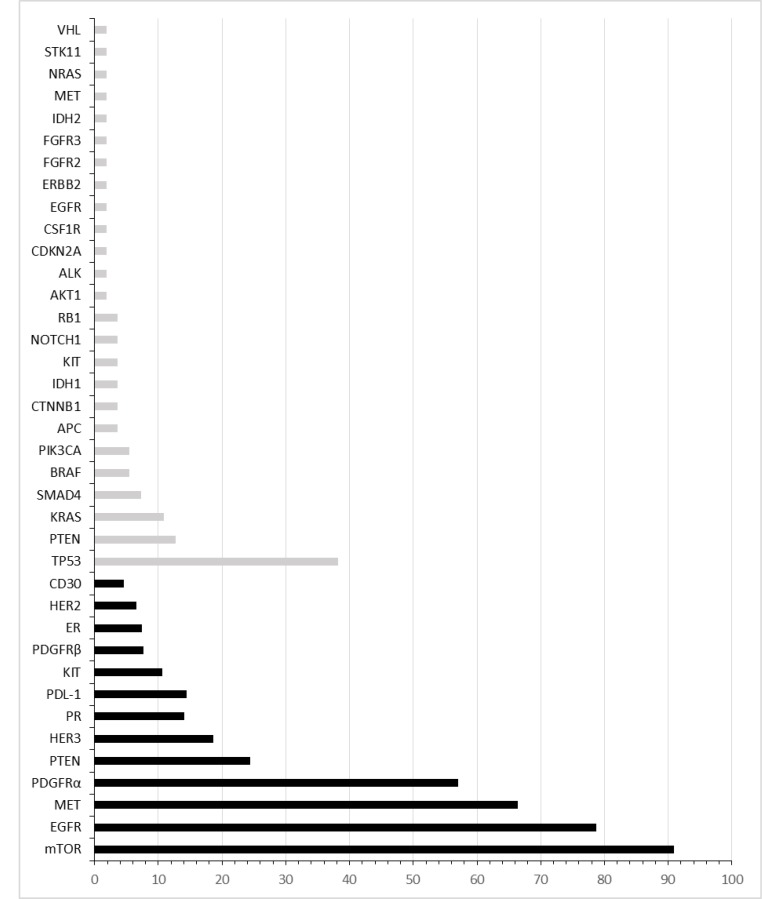
Molecular profile assessed by immunohistochemistry and next generation sequencing All immunoreactivity positive cases (black bars) and patients with genetic mutations (grey bars) are represented as percentage of enrolled subjects.

Tumor samples of 55 patients were real-time analyzed via NGS, IHC and FISH using a platform of a parallel study (unpublished data). The most frequent somatic mutations observed were in TP53 (38%), PTEN (13%) and KRAS (11%). Frequently observed increases in protein expression as assessed by IHC were EGFR (75%), phospho-mTOR (75%), MET (69%) and PDGFRa (55%) (Figure 1; for histochemistry examples refer to Figure S1). FISH was performed, but FISH results alone did not lead to treatment suggestions (data not shown).

### Response rate

Patients were treated with kinase inhibitors, growth factor receptor antibodies in combination with hormone therapy or chemotherapy or were given immunotherapy (Table 2). The median PFS1/PFS0 ratio of all patient was 1.35 (quartile 0.7/2.9). From 55 patients, 34 (62%) showed a PFS1/PFS0 ratio >1.0 under the experimental therapy (PFS1) (*p =* 0.002) (Table 4). Thus, the primary study aim was met as the null hypothesis was rejected.

**Table 2:** Treatment of 34 patients (62%) based on molecular profiling with PFS1/PFS0 >1.0°

Tumor^§^	Genetic profile	IHC	Therapy	Evidence supporting treatment decision	
CUP	Atypical ALK rearrangement	MET+, PR+	Crizotinib Tamoxifen	[19, 20]	
CUP	KITmutation	KIT+	Imatinib	[21]	
CUP		mTOR+	Everolimus	[22, 23]	
Thyroid	BRAF mutation (V600E)		Vemurafenib	[24]	
Thyroid		mTOR+ EGFR+	Temsirolimus Cetuximab	[25, 26]	
Thyroid		PDL-1+	Pembrolizumab	[27, 28]	
CCC		EGFR+ (RAS wildtype)	Irinotecan Cetuximab	[29]	
CCC		EGFR+ (RAS wildtype)	Irinotecan Cetuximab	[29]	
CCC		EGFR+ (RAS wildtype)	Irinotecan Cetuximab	[29]	
CCC	FGFR2 mutation	MET+	Regorafenib	[30, 31]	
HCC		EGFR+	Irinotecan Cetuximab	[32, 33]	
HCC		EGFR+	Folfox Cetuximab	[34]	
GEC		HER2+	Trastuzumab Pertuzumab	[35]	
GEC		HER2+	Folfiri Trastuzumab	[35]	
CRC		PDL1– MSHI high	Pembrolizumab^*^	[36-38]	
CRC		MSHI high	Pembrolizumab^*^	[36-38]	
CRC		MET+ mTOR+	Temsirolimus Bevacizumab beyond progression	[26]	
CRC	KIT mutation	MET+	Sunitinib	[39]	
RRC		EGFR+ HER2+ HER3+	Trastuzumab Lapatinib	[40]	
Prostate		mTOR+	Temsirolimus	[25, 41]	
H&N		PDL-1+	Pembrolizumab^*^	[42, 43]	
H&N		mTOR+	Carboplatin Everolimus	[44, 45]	
H&N		PDGFRa+	Docetaxel Sunitinib	[46]	
NHL		mTOR+	Everolimus	[22, 23]	
NHL		CD30+	Brentuximab		[47]
Ovarian		ER+ PR+ PDGFa+ mTOR+	Letrozol Bevacizumab	[48, 49]	
Myeloma		PDL-1+	Pembrolizumab	[50]	
SCLC	EGFR mutation	EGFR +	Afatinib	[51]	
Pleuramesothelioma		PDL-1 +	Pembrolizumab	[52]	
Pleuramesothelioma		PDGFRa+ PDGFRb+	Sunitinib	[39, 53]	
Pleuramesothelioma		PDGFRb+	Palbociclib	[54]	
PEComa		PDGFRa+ PDGFRb+ EGFR+	Sunitinib	[39, 55]	
Endometrial		ER+ mTOR+	Exemestan Everolimus	[56]	
Vulva		PDL-1+	Pembrolizumab	[52]	

^§^CUP: cancer of unknown primary, CCC: cholangiocarcinoma, HCC: hepatocellular carcinoma, GEC: gastroesophageal cancer, CRC: colorectal cancer, RRC: renal cancer, H&N: head and neck cancer, SCLC: small cell lung cancer, NHL: non Hodgkin lymphoma, PEComa: perivascular epithelioid cell tumor *of the* kidney.

°The cut-offs values for the selection of putative druggable targets were determined as follows: PDL-1: presence of positive tumor cells, Tumor Proportion Score ≥1, mTOR: IHC score: 200–300, HER2: score ≥2 and confirmed amplification by FISH, KIT: IHC Score 100–300, PR: Allred Score ≥6, EGFR: IHC score 200–300, PDGFRα: IHC score 100–300, PDGFRβ: IHC score 200–300, ER: Allred Score ≥3, CD30: % of positive lymphoma cells, MET: IHC Score ≥2+ and HER3: IHC Score 100–300.

^*^At the time of treatment decision, pembrolizumab was not approved by neither the (U. S. Food and Drug Administration) FDA nor the European Medicines Agency (EMA).

**Table 3:** Treatment response rate upon experimental therapy

Treatment response	Number of patients	% of total
CR	2	4
PR	14	26
SD	15	27
PD	21	38
Ongoing	3	5
ORR	16	29
DCR	31	56
Total (*n*, %)	55	100

CR: complete remission; PR: partial remission; SD: stable disease; PD: progressive disease; ORR: overall response rate (patients with complete or partial remissions); DCR: disease control rate (patients with complete remission, partial remission or stable disease).

**Table 4:** Survival data

Parameter	Median	Quartile
OS	348	177/664
PFS 0	61	51/92
PFS 1	112	66/201
Ratio PFS1/PFS0	1.35	0.7/2.9^*^

Quartiles cover the 25% and 75% range; ^*^for the ratio PFS1/PFS0, the range (min/max) is displayed.

**Figure 2:**
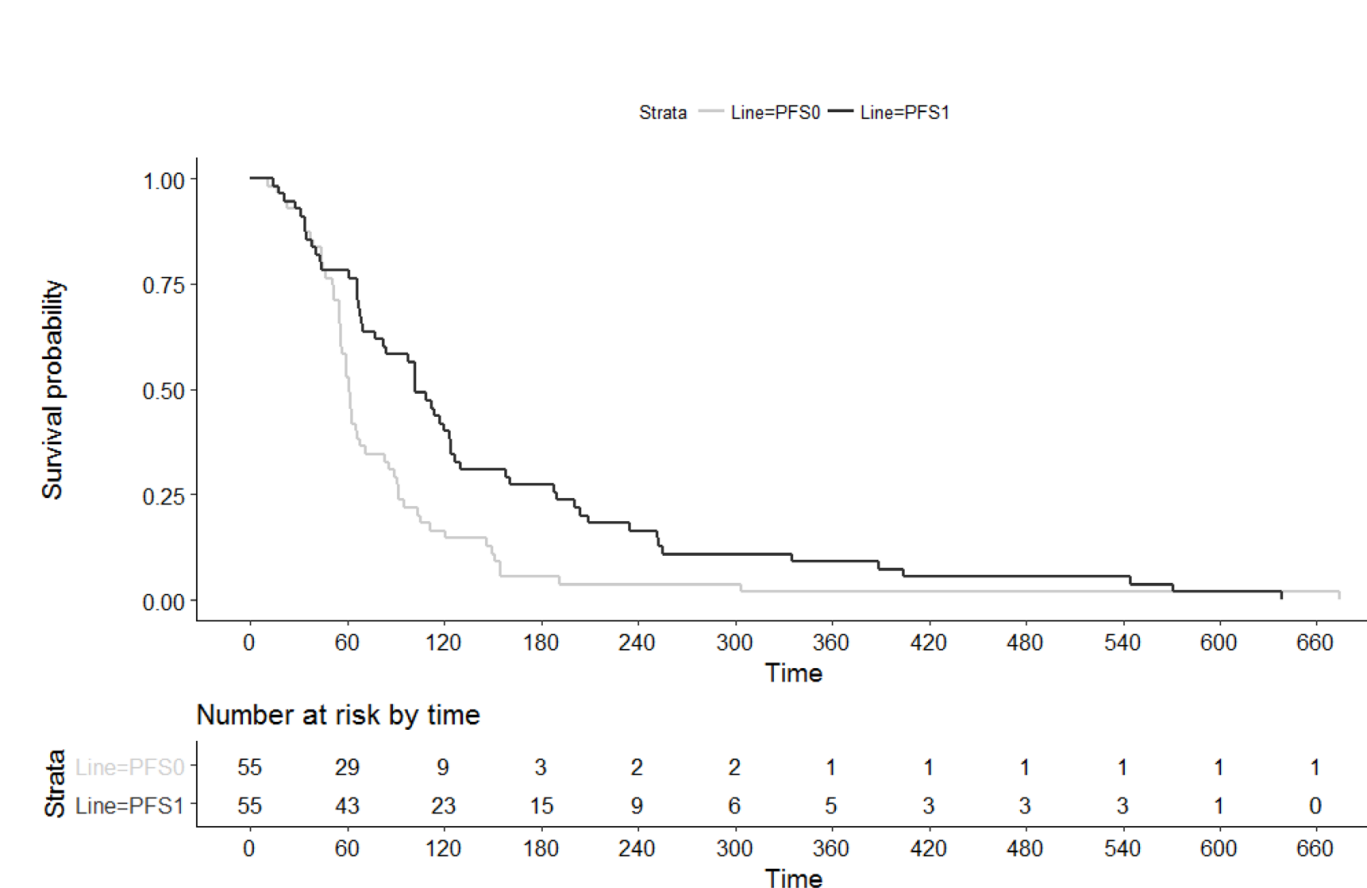
Progression free survival upon last standard therapy (PFS0) and experimental individualized therapy (PFS1)

29% of patients (*n =* 16) showed an overall response according to RECIST. The disease control rate was 56% (*n =* 31). Out of 55 patients, 2 (4%) did show a complete remission and 14 patients (25%) had a partial remission while 15 patients (27%) had a stable disease according to RECIST 1.1 criteria (Table 3). 38% (*n* = 21) did not benefit from therapy and were progressive. Note that three patients were still under experimental therapy and were not evaluated for treatment response at the day of censoring (Figure 2).

### Survival data

The median PFS of the individualized treatment (PFS1) was 112 days (quartiles 66/201). The median PFS of the therapy given before (PFS0) was 61 days (quartiles 51/92). The overall survival at the day of censoring was 348 days (quartiles177/664). In the Wilcoxon Signed Rank Test PFS1 was significantly longer than PFS0 (*p =* 0.002). The 95% bootstrap confidence interval of the median of the ratio PFS1/PFS0 is [1.086; 2.034].

## DISCUSSION

In this study we present a prospective clinical phase II trial to determine efficacy of individualized therapy. Tissue derived from real-time biopsies of 55 patients suffering from refractory cancer was characterized for their molecular profile and individualized treatment was suggested by a multidisciplinary team. From the 114 patients tested, 55 (48%) started treatment according to their tumor’s molecular profile. The median PFS under experimental treatment (PFS1) was 112 days and was significant longer than the median PFS upon the previous treatment. Notably and even more important, on the individual base, 34 (62%) patients achieved a longer PFS than upon the previous treatment, thus, the null hypothesis was clearly rejected. Thus, the majority of the patients had a clinical benefit from this individualized treatment concept in a late line setting. Furthermore, at time of censoring the median overall survival was 348 days (quartiles 177/664).

The ability to identify driver mutations in tumors had led to the introduction targeted treatments interfering with these tumor drivers. Although certain driver mutation are not tumor type specific, targeted treatments have so far been approved by authorities rather by origin of the tumor than by its mutation. In this context, the example of BRAF inhibitory drugs have been shown to be active in BRAF V600 mutated tumors [11], but when starting EXACT, approval of these drugs was limited to BRAF mutated melanoma. That BRAF inhibition might be effective in BRAF mutated tumors outside of melanomas is supported in this study. To address a potential biological activity of targeted drugs in rare mutations, so called basket trials are addressing a certain molecular mutation to assess whether a treatment concept is similarly amenable to identical targeted treatment. Other actionable biomarkers such as PD-L1 expression are thought to be less sensitive, but might be used as stratification factors in clinical trials. As somatic tumor mutations are amended to instability, real-time biopsy seems to be adequate for patients in treatment-refractory cancers [12–16].

In a meta-analysis, it was demonstrated that this concept has been achieved best results for efficacy of precision medicine in phase II trials [17]. This is consistent with our own observations of clonal selection during previous treatment and support the conclusion that targeted treatment should be exclusively suggested upon molecular profiling of real-time biopsies rather than from archival tissue.

In one of the first individualized treatment studies in cancer, von Hoff *et al.* reported beneficial effects of molecular profiling of patient`s tumors [16]. In this particular study 27% of the patients had a benefit from an individual target approach by resulting in a significant longer PFS when compared with the PFS of the previous regimen of the same patient (PFS ratio ≥ 1.3; 95% CI, 17% to 38%; one-sided, one-sample *P =* .007). Although a cross over between trials is not possible, our results of 58% of patients demonstrated an increased PFS under targeted treatment is stimulating. This might partially explained by the fact of progress in diagnostic techniques and therapeutic options within the last few years. In fact, we were considering beside other options checkpoint inhibitors as potential treatment options.

By recent advances of targeted treatment studies it becomes mandatory to evaluate potential effectiveness of marketed, targeted anticancer drugs when applied outside of their approved indications. In this context, ASCO is developing the Targeted Agent and Profiling Utilization Registry (TAPUR) study, which aims to facilitate patient access to marketed agents that are predicted to be beneficial based on analysis of patients’ tumor’s genomic profile. Furthermore, and most importantly, by capturing their outcomes in a prospective database this approach will improve the understanding which treatment concepts might lead to patient benefit. TAPUR is thereby conceptually similar to both the ongoing major initiative of the U.S. NCI, the Molecular Analysis for Therapy Choice (MATCH) trial as well as the AcSé program being conducted by the French National Cancer institute.

Although our study bears some weaknesses such as the fact that it was not randomized and the sample size was small, we feel it is important to emphasize that molecular profiling- based treatment decisions is feasible for a subgroup of patients to improve their prognosis when no standard treatment options are available. The use of a matched control arm to evaluate the benefit of molecular profiling in pretreated cancer patients is urged and the focus of a consecutive trial. With increasing number of new targeted treatment options available, the concept of an individualized therapy will likely be in focus of future treatment concepts in cancer.

## MATERIALS AND METHODS

The study was conducted in accordance with the Declaration of Helsinki and was approved by the institutional ethic committee of the Medical University of Vienna (Nr.1541/2012).

### Study design

The primary objective of the study was to prospectively validate the benefit of an individualized treatment concept based on molecular profiling from paraffin-embedded tumor tissue sections obtained before the start of treatment (real time biopsy).

Rejection of the null hypothesis was defined as follows: ≤40% of this patient population would have a PFS ratio of > 1.0. Thus, the individual patient served as his own control. The alternative proportion P1 (PFS ratio > 1.0) is set at least to 55% using a one-sided exact binomial test at a significance level of 0.0250. The null hypothesis can be rejected, if at least 30 out of 55 patients treated show a PFS ratio >1.0.

Furthermore, treatment response (ORR) and overall survival (OS) was assessed by by CT scans at 8-week intervals during therapy and judged according to the Response Evaluation Criteria in Solid Tumors (RECIST), version 1.1 and evaluated as secondary end points.

### Patients

Patient eligibility criteria included informed consent, any histologic type of metastatic cancer without further standard treatment option, tumor progression by RECIST criteria, age ≥ 18 years, ECOG performance status 0-1. Fresh tumor biopsy was obtained for pathologic analysis. Biopsies were performed by a heterogeneous group of different surgical techniques routinely used at the Department of Interventional Radiology. Patients were thought to be eligible if treatment could be initiated upon the molecular profile derived from real-time biopsy (see Supplementary Figure 2).

### Tissue samples

Tissues from metastatic cancer patients were formalin fixed and paraffin embedded at the Department of Pathology, Medical University Vienna. for histology and molecular diagnostics.

### Cancer gene panel sequencing

The DNA library was generated by multiplex polymerase chain reaction (PCR) with the Ion AmpliSeq Cancer Hotspot Panel v2™ (Life Technologies, Carlsbad, CA). The panel covers mutation hotspots of 50 genes, mostly oncogenes and tumor suppressor genes that are frequently mutated in tumors (ABL, AKT, ALK, APC, ATM, BRAF, CDH, CDKN2A, CSF1R, CTNNB1, EGFR, ERBB2, ERBB4, EZH2, FBXW7, FGFR1, FGFR2, FGFR3, FLT3, GNA11, GNAS, GNAQ, HNF1A, HRAS, IDH1, JAK2, JAK3, IDH2, KDR, KIT, KRAS, MET, MLH1, MPL, NOTCH1, NPM1, NRAS, PDGFRA, PIK3CA, PTEN, PTPN11, RB1, RET, SMAD4, SMARCB1, SMO, SRC, STK11, TP53, VHL). Sequencing was performed with an Ion Torrent PGM™ (Life Technologies). Nonsynonymous mutations detected with the Ion Torrent PGM™ were verified by capillary sequencing. The sequencing of PCR products was carried out with the BigDyeR Terminator v1.1 Cycle Sequencing Kit (Applied Biosystems, Waltham, MA). The resulting DNA fragments were purified with the DyeEx 96 Kit (Qiagen) and sequenced with a 3500 Genetic Analyzer (Applied Biosystems). For sequence analysis we employed the SeqScape Version 2.7 software (Applied Biosystems).

### Immunohistochemistry

Immunohistochemistry was performed with a Ventana Benchmark Ultra stainer (Ventana, Tucson, AZ). The following antibodies were employed: ALK (clone 1A4; Zytomed, Berlin, Germany), CD30 (clone BerH2; Dako, Vienna, Austria), CD20 (clone L26; Dako), EGFR (clone 3C6; Ventana), Estrogen-receptor (clone SP1; Ventana), HER2 (clone 4B5; Ventana), HER3 (clone SP71; Abcam), KIT (clone 9.7; Ventana), MET (clone SP44; Ventana), phospho-mTOR (clone 49F9; Cell Signalling, Danvers, MS), PDGFRA (rabbit polyclonal; Thermo Fisher Scientific), PDGFRB (clone 28E1, Cell Signalling), PD-L1 (clone E1L3N; Cell Signalling), Progesteron-receptor (clone 1E2; Ventana), PTEN (clone Y184; Abcam) and ROS1 (clone D4D6; Cell Signalling). For details refer to the Supplementary Files (Supplementary Table 1).

The diagnostic sensitivity and specifity of the antibodies has been validated at the Department of Pathology at the Medical University Vienna. For the validation appropriate positive and negative tissue controls were employed (see Supplementary Figure 1). Furthermore, the omission of primary antibodies and the replacement of primary antibodies by antibodies of the same species, isotype and concentration, having no known reactivity against human tissue, served as negative reagent controls. The antibodies employed in this study have been institutionally approved for the application in routine histopathological diagnostics. The antibodies to ALK, CD30, EGFR, HER2 and MET are additionally licensed *in vitro* diagnostics, the antibody to CD20 is CE marked.

For the evaluation of staining intensities with antibodies to EGFR, phospho-mTOR, PDGFRA, PDGFRB and PTEN an immunohistochemial score was determined by multiplying the percentage of positive cells by their respective staining intensity (0 = negative, 1 = weak, 2 = moderate, 3 = strong). Immunohistochemical score (maximum 300) = (% negative × 0) + (% weak × 1) + (% moderate × 2) + (% strong × 3).

ALK, CD30, CD20 and ROS1 stainings were categorised as positive or negative with the percentage of reactive neoplastic cells, but without scoring of staining intensities. ALK or ROS1 positive cases were consecutively interrogated for the presence of a respective gene translocation by FISH. HER2 staining was graduated according to the guidelines of the company Dako for the Dako HercepTest^R^ with possible scores 0 (negative), 1+ (negative), 2+ (positive), 3+ (positive). HER2 2+ cases were further analysed by HER2 *in-situ* hybridization to verify a HER2 gene amplification. For PD-L1 the percentage of tumor cells with a membranous staining, irrespective of staining intensity, was determined (so-called “tumor proportion score”). MET staining was graduated according to a published scoring system that evaluated both staining intensity (negative, weak, moderate, or strong) and prevalence of these intensities in tumor cells [18].

### Fluorescence *in situ* hybridization (FISH)

FISH was performed with 4 μm thick FFPE tissue sections. The following FISH probes were employed: ALK (2p23.1; Abbott, Abbott Park, IL), RET (10q11; Kreatech, Berlin, Germany), PTEN (10q23.31)/Centromer 10) and ROS1 (Zytovision, Bremerhaven, Germany). 200 cell nuclei per tumor were evaluated. To detect HER2, two diagnostic systems were applied: FISH (PathVysion II; Abbott) and CISH (Ventana Medical Systems by Roche Diagnostics).

### Treatment algorithm

Patients with refractory metastatic cancer without any standard treatment options according to NCCN guidelines and/or local guidelines were included. Potential therapeutic targets in individual patient’s tumor sections were individualized by genomic tumor profiling (NGS and FISH) in combination with immunohistochemistry. The generated data were biostatistically combined with the actual data from clinical trials thus resulting in the identification of druggable targets (drivers) with the highest likelihood of response in each individual patient. Based on these recommendations, previous lines of treatment and patient performance status, final treatment decisions were made by at least two different medical oncologists in a molecular tumor board with participation of pathologists, radiologists and translational scientists. Only agents with marketing authorization and established safety profile for combinations were used in off-label settings in this trial.

### Statistics

For statistical analysis whether PFS0 and PFS1 are different, a Wilcoxon Signed Rank Test was performed. To find possible patient characteristics that influence the difference between PFS0 and PFS1 a linear model was performed with the difference (PFS1 - PFS0) as dependent, and age as well as gender as independent variables. Furthermore a 95% confidence interval for the median of the ratio PFS1/PFS0 was calculated through bootstrap with 1000 samples. Note that three of the 55 patients had still an ongoing therapy, but were treated as if the therapy has already ended. Analysis was performed using statistical Software R Version 3.3.0.

## SUPPLEMENTARY MATERIALS FIGURES AND TABLE


