# Supplementary material for: Results of the extended analysis for cancer treatment (EXACT) trial: a prospective translational study evaluating individualized treatment regimens in oncology
Source: Oncotarget. 2019 Jan 29;10(9):942–52. doi: 10.18632/oncotarget.26604 (PMC6398177; doi:10.18632/oncotarget.26604)
Supplement: Supplementary file 1 [file oncotarget-10-942-s001.pdf]

Results of the extended analysis for cancer treatment (EXACT) trial: a prospective translational study evaluating individualized treatment regimens in oncology

SUPPLEMENTARY MATERIALS

Immunohistochemistry

Immunohistochemistry was done with 2 µm thin FFPE tissue sections. The used primary antibodies are described in Table 1. The immunohistochemistry was performed with a Ventana Benchmark Ultra stainer (Ventana, Tucson, AZ) with extended heat-induced epitope rerieval with CC1 buffer and the ultraView Universal DAB Detection Kit (Ventana) or with a Bond III stainer (Leica Biosystems, Vienna, Austria) for CD20 and CD30.

The antibodies were either CE and/or IVD marked or for research use only and validated at the Institute of Pathology at the Medical University Vienna for diagnostic use with appropriate positive and negative tissue controls and isotype-matched control antibodies.

Fluorescence *in-situ* hybridisation (FISH)

FISH was performed with 4 µm thick FFPE tissue sections. The following FISH probes were employed: ALK (2p23.1; Abbott, Abbott Park, IL), RET(10q11; Kreatech, Berlin, Germany), PTEN (10q23.31)/Centromer 10) and ROS1 (Zytovision, Bremerhaven, Germany). 200 cell nuclei per tumor were evaluated. To detect HER2, two diagnostic systems were applied: FISH (PathVysion II; Abbott) and CISH (Ventana Medical Systems by Roche Diagnostics).

Supplementary Table 1: Antibodies used for immunohistochemistry

Antibody	Clone	Supplier	Regulatory status	Antibody Dilution	Scoring	Cut-off	Reference
ALK	1A4	Ventana	IVD@	1:50	IHC score*	Presence of reactive neoplastic cells (IHC score >0) and translocation by FISH	[1]
CD20	L26	Dako	CE-IVD+	1:400	% positive tumor cells	Percentage of reactive neoplastic cells	[2]
CD30	BERH2	Dako	CE-IVD	1:50	% positive tumor cells	Percentage of reactive neoplastic cells	[3]
EGFR	3C6	Ventana	IVD	prediluted - ready to use	IHC score*	IHC score 200-300	[4]
Estrogen-receptor	SP1	Ventana	IVD	prediluted - ready to use	Allred Score, ref. (5)	Allred Score ≥ 3	[6]
HER2	4B5	Ventana	FDA##, IVD	prediluted - ready to use	Scoring scale of 0 to 3+ according to guidelines described by Ventana#	Score ≥ 2 and confirmed amplification by CISH and/or FISH	[7]
HER3	SP71	Abcam	RUO$	1:100	IHC score*	IHC Score 100-300	[8]
KIT	9.7	Ventana	CE	prediluted - ready to use	IHC score*	IHC Score 100-300	[9]
MET	SP44	Ventana	IVD	prediluted - ready to use	Four subgroups, according to ref. (10)	Score ≥ 2+	[11]
mTOR	49F9	Cell Signalling Technology	RUO	1:50	IHC score*	IHC Score 100–300 and PTEN loss	[12]
PDGFRA	rabbit polyclonal	Thermo Fisher Scientific	RUO	1:50	IHC score*	IHC score 100–300	[13]
PDGFRB	28E1	Cell Signalling Technology	RUO	1:50	IHC score*	IHC score 200–300	[13]
PD-L1	E1L3N	Cell Signalling Technology	RUO	1:50	% positive tumor cells	Presence of reactive neoplastic cells ≥ 1	[14]
Progesteron-receptor	1E2	Ventana	IVD	prediluted - ready to use	Allred Score, ref. [5]	Allred Score ≥ 6	[15]
PTEN	Y184	Abcam	IVD	1:50	IHC score*	IHC Score 0	[16]
ROS1	D4D6	Cell Signalling Technology	RUO	1:50	IHC score*	Presence of reactive neoplastic cells (IHC score >0) and translocation by FISH	[17]

*An immunohistochemial score (IHC score) was determined by multiplying the percentage of positive cells by their respective staining intensity (0 = negative, 1 = weak, 2 = moderate, 3 = strong). Immunohistochemical score (maximum 300) = (% negative × 0) + (% weak × 1) + (% moderate × 2) + (% strong × 3).

Equivocal 2+ staining cases were additionaly analysed by DNA in-situ hybridisation to validate for HER2 gene amplification

@ For in vitro diagnostic use

+ CE, certification mark that indicates conformity with health, safety, and environmental protection standards for products sold within the European Economic Area (EEA).

FDA## US Food and Drug Administration approved

$ for research use only

REFERENCES

1. Wang Q, Zhao L, Yang X, Wei S, Zeng Y, Mao C, Lin L, Fu P, Lyu L, Li Z, Xiao H. Antibody 1A4 with routine immunohistochemistry demonstrates high sensitivity for ALK rearrangement screening of Chinese lung adenocarcinoma patients: A single-center large-scale study. Lung Cancer. 2016; 95:39–43.
2. Mason DY, Comans-Bitter WM, Cordell JL, Verhoeven MA, van Dongen JJ. Antibody L26 recognizes an intracellular epitope on the B-cell-associated CD20 antigen. The American journal of pathology. 1990; 136:1215–22.
3. Schwarting R, Gerdes J, Durkop H, Falini B, Pileri S, Stein H. BER-H2: a new anti-Ki-1 (CD30) monoclonal antibody directed at a formol-resistant epitope. Blood. 1989; 74:1678–89.
4. Mascaux C, Wynes MW, Kato Y, Tran C, Asuncion BR, ZhaoJM, Gustavson M, Ranger-Moore J, Gaire F, Matsubayashi J, Nagao T, Yoshida K, Ohira T, et al. EGFR Protein Expression in Non-Small Cell Lung Cancer Predicts Response to an EGFR Tyrosine Kinase Inhibitor – A Novel Antibody for Immunohistochemistry or AQUA Technology. Clin Cancer Res. 2011; 17:7796–807.
5. Fitzgibbons PL, Dillon DA, Alsabeh R, Berman MA, Hayes DF, Hicks DG, Hughes KS, Nofech-Mozes S. Template for reporting results of biomarker testing of specimens from patients with carcinoma of the breast. Arch Pathol Lab Med. 2014; 138:595–601.
6. Welsh AW, Harigopal M, Wimberly H, Prasad M, Rimm DL. Quantitative Analysis of Estrogen Receptor Expression Shows SP1 antibody is more sensitive than 1D5. Appl Immunohistochem Mol Morphol. 2013; 21:139–47.
7. Ruschoff J, Dietel M, Baretton G, Arbogast S, Walch A, Monges G, Chenard MP, Penault-Llorca F, Nagelmeier I, Schlake W, Höfler H, Kreipe HH. HER2 diagnostics in gastric cancer-guideline validation and development of standardized immunohistochemical testing. Virchows Arch. 2010; 457:299–307.
8. Lédel F, Hallström M, Ragnhammar P, Öhrling K, Edler D. HER3 expression in patients with primary colorectal cancer and corresponding lymph node metastases related to clinical outcome. Eur J Cancer. 2014; 50:656–62.
9. Hornick JL, Fletcher CD. Immunohistochemical staining for KIT (CD117) in soft tissue sarcomas is very limited in distribution. Am J Clin Pathol. 2002; 117:188–93.
10. Spigel DR, Ervin TJ, Ramlau RA, Daniel DB, Goldschmidt JH Jr, Blumenschein GR Jr, et al. Randomized phase II trial of Onartuzumab in combination with erlotinib in patients with advanced non-small-cell lung cancer. J Clin Oncol. 2013; 31:4105–14.
11. Dziadziuszko R, Wynes MW, Singh S, Asuncion BR, Ranger-Moore J, Konopa K, Rzyman W, Szostakiewicz B, Jassem J, Hirsch FR. Correlation between MET Gene Copy Number by Silver in Situ Hybridization and Protein Expression by Immunohistochemistry in Non-Small-Cell Lung Cancer. J Thorac Oncol. 2012; 7:340–7.
12. Leal P, Garcia P, Sandoval A, Letelier P, Brebi P, Ili C, Álvarez H, Tapia O, Roa JC. Immunohistochemical expression of phospho-mTOR is associated with poor prognosis in patients with gallbladder adenocarcinoma. Arch Pathol Lab Med. 2013; 137:552–7.
13. Bergsten E, Uutela M, Li X, Pietras K, Ostman A, Heldin CH, et al. PDGF-D is a specific, protease-activated ligand for the PDGF beta-receptor. Nat Cell Biol. 2001; 3:512–6.
14. Sholl LM, Aisner DL, Allen TC, Beasley MB, Borczuk AC, Cagle PT, Capelozzi V, Dacic S, Hariri L, Kerr KM, Lantuejoul S, Mino-Kenudson M, Raparia K, et al. Programmed Death Ligand-1 Immunohistochemistry--A New Challenge for Pathologists: A Perspective From Members of the Pulmonary Pathology Society. Arch Pathol Lab Med. 2016;140:341-4.
15. Ibrahim M, Dodson A, Barnett S, Fish D, Jasani B, Miller K. Potential for false-positive staining with a rabbit monoclonal antibody to progesterone receptor (SP2): findings of the UK National External Quality Assessment Scheme for Immunocytochemistry and FISH highlight the need for correct validation of antibodies on introduction to the laboratory. Am J Clin Pathol. 2008; 129:398–409.
16. Akesson E, Gallos ID, Ganesan R, Varma R, Gupta JK. Prognostic significance of estrogen and progesterone receptor expression in LNG-IUS (Mirena) treatment of endometrial hyperplasia: an immunohistochemical study. Acta Obstet Gynecol Scand. 2010; 89:393–8.
17. Sholl LM, Sun H, Butaney M, Zhang C, Lee C, Janne PA, et al. ROS1 immunohistochemistry for detection of ROS1-rearranged lung adenocarcinomas. Am J Surg Pathol. 2013; 37:1441–9.

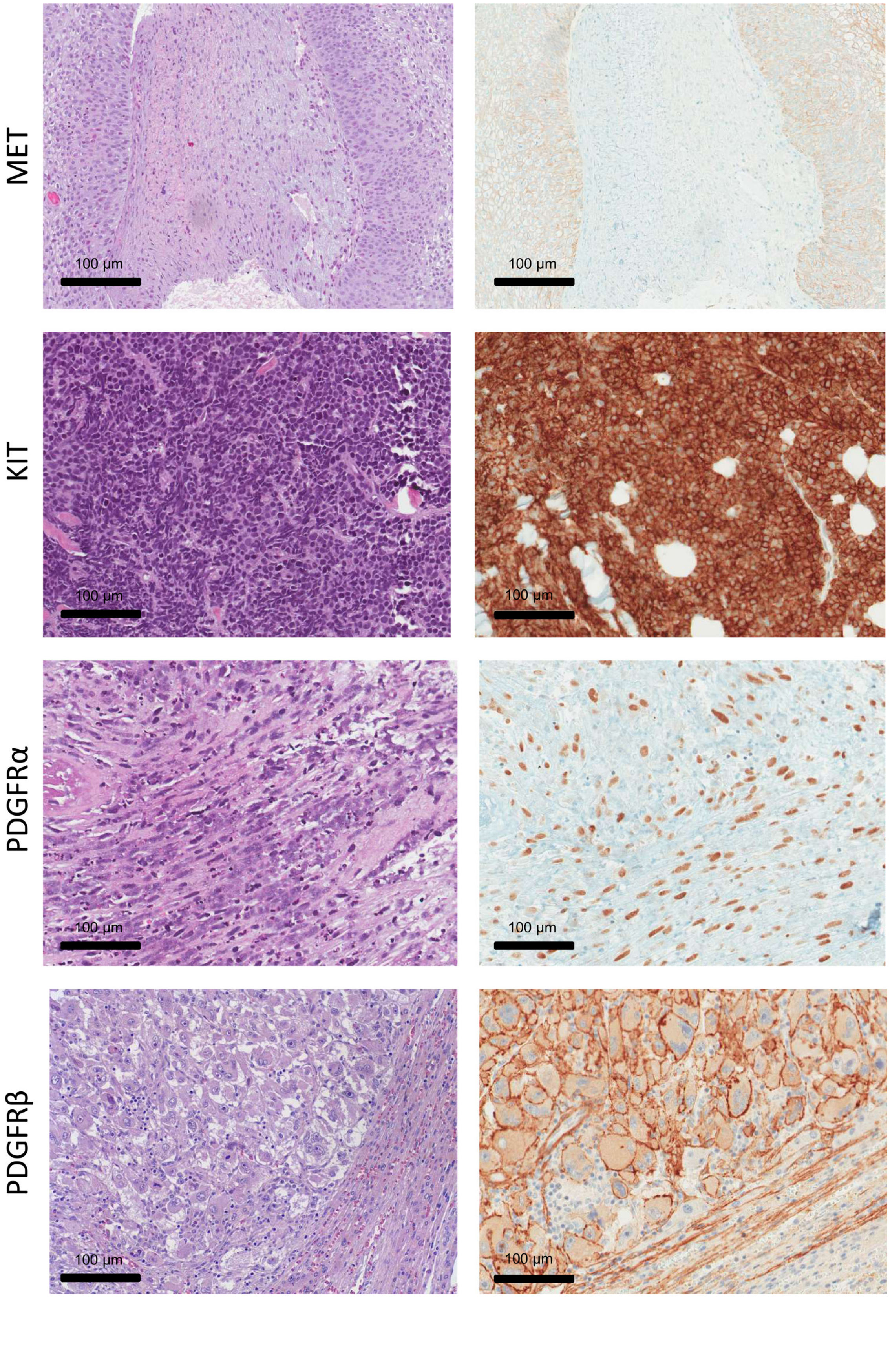
MET
KIT
PDGFRα
PDGFRβ
100 µm

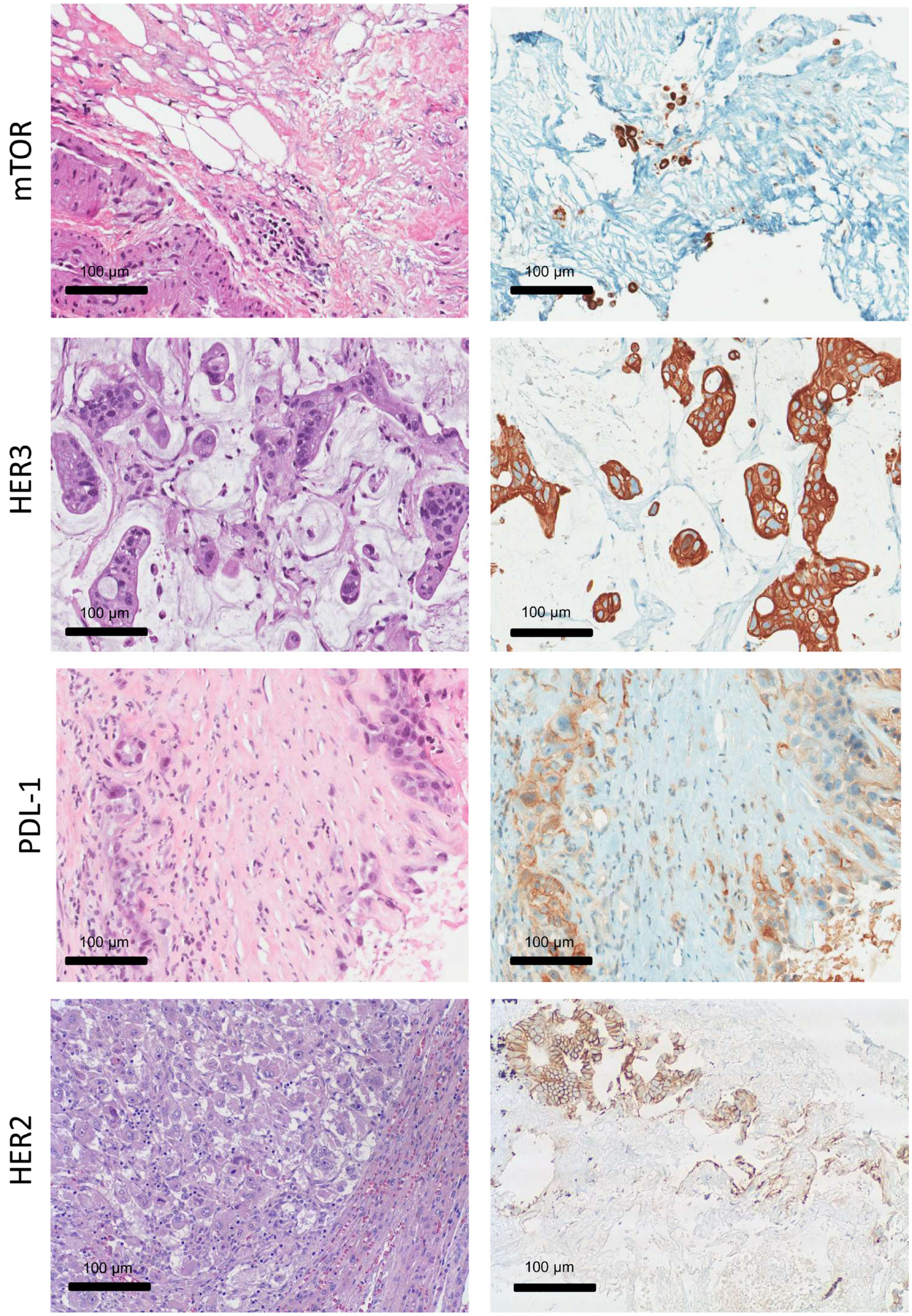
mTOR
HER3
PDL-1
HER2
100 µm

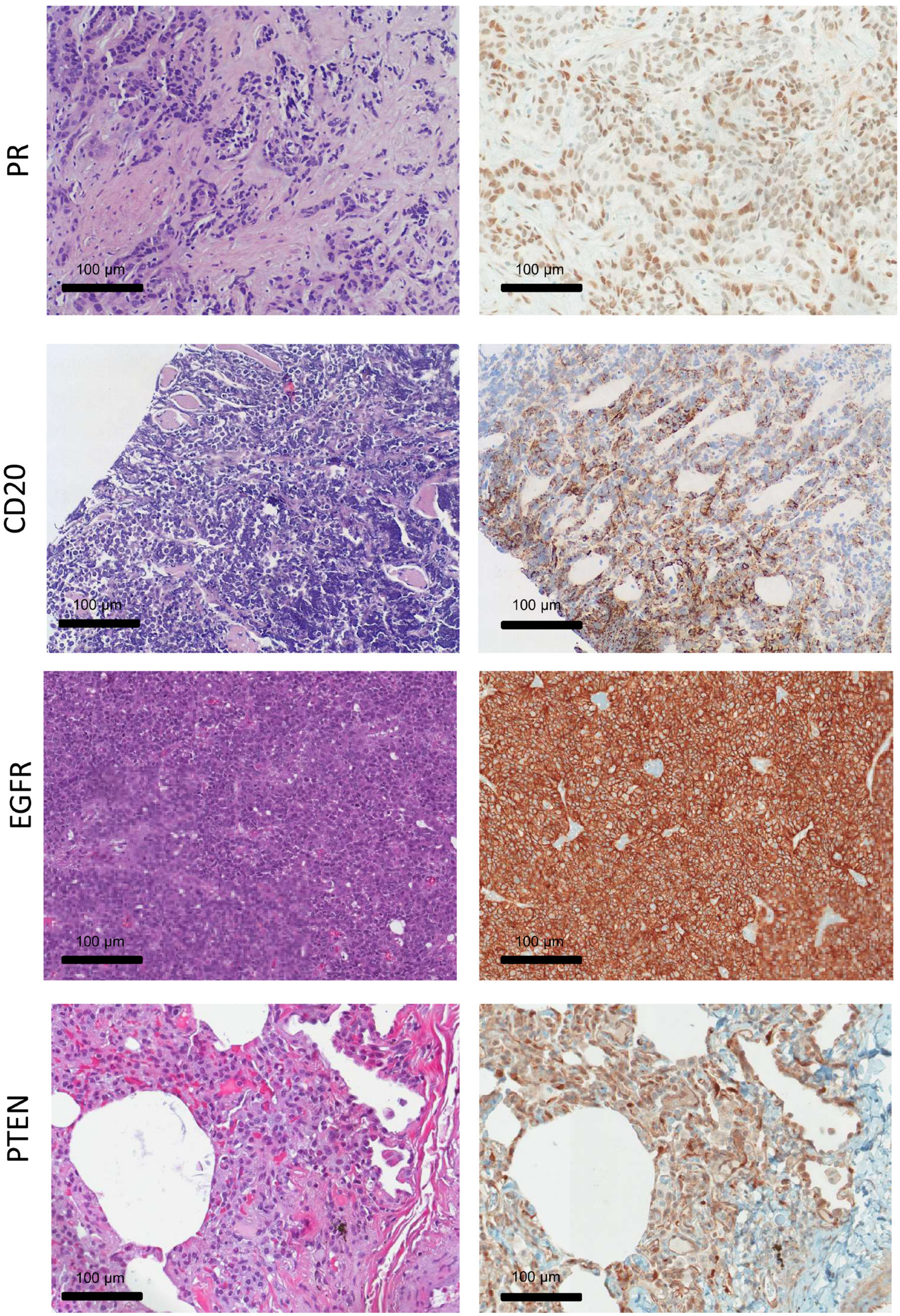
PR
CD20
EGFR
PTEN
100 µm

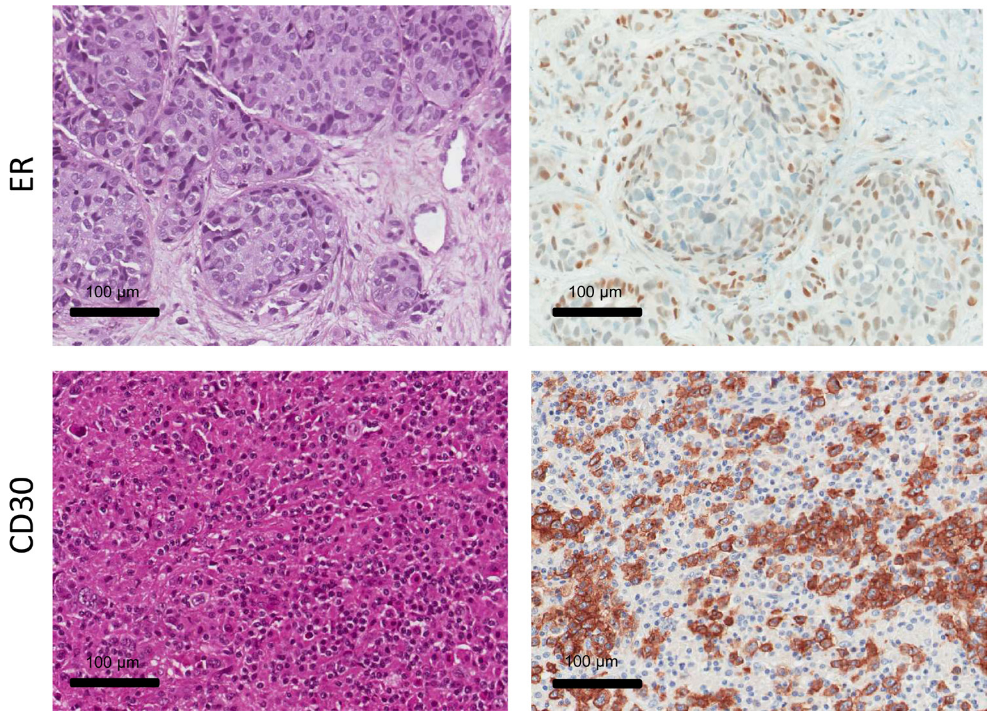

Supplementary Figure 1: Representative images of hematoxylin and eosin staining (on the left) and immunohistochemical staining of tumors (on the right). The scoring of the depicted immunoreactivity positive cases are:MET: 3+; KIT: IHC Score 300; PDGFRα: IHC Score 200, PDGFRβ: IHC Score 300; mTOR: IHC Score 99, HER3: IHC Score 300, PDL-1: 85%; HER2: 2+; PR: Allred Score 6; CD20: 95%; EGFR: IHC Score 300, PTEN: IHC Score 250; ER: Allred Score 3 and CD30: 95%

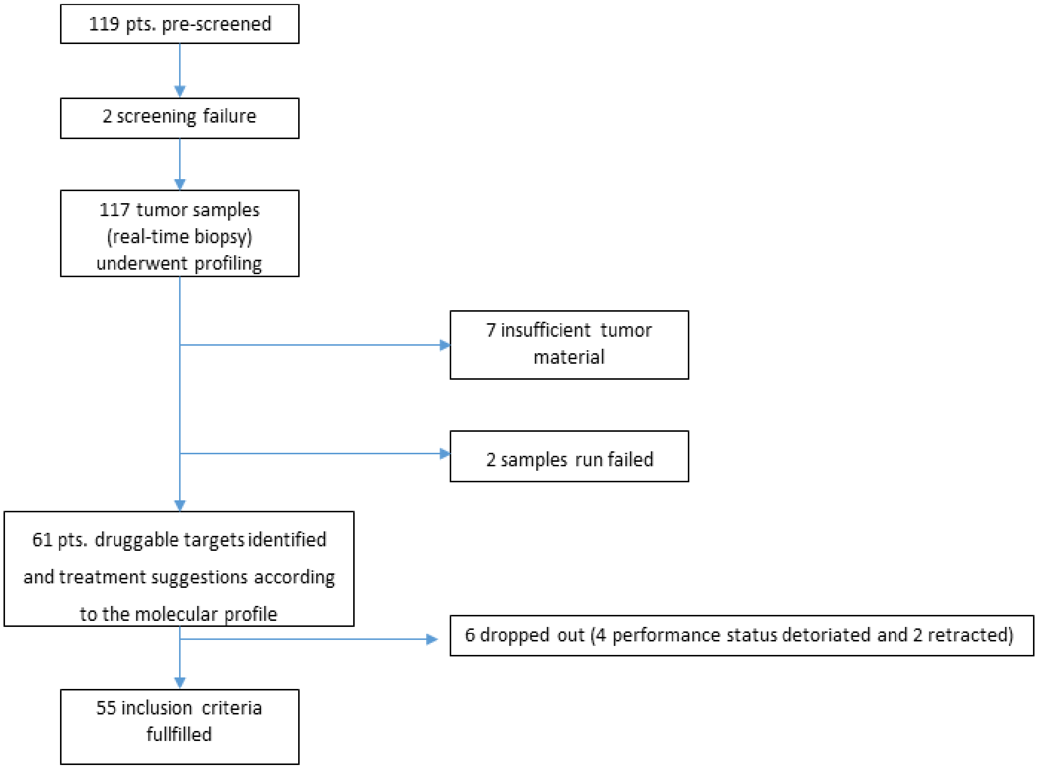

Supplementary Figure 2: Flowchart of patients pre-screened for the EXACT trial; from 119 patients (pts.) pre-screened, in 61 patients druggable targets were identied and in 55 experimental treatment according to the respective molecular profile was initiated within the EXACT-trial, thereby fullfilling the inclusion criteria.
